# Concepts for augmented reality visualisation to support needle guidance inside the MRI

**DOI:** 10.1049/htl.2018.5076

**Published:** 2018-09-18

**Authors:** André Mewes, Florian Heinrich, Bennet Hensen, Frank Wacker, Kai Lawonn, Christian Hansen

**Affiliations:** 1 Faculty of Computer Science, Otto-von-Guericke University Magdeburg, Germany; 2 Research Campus STIMULATE, Otto-von-Guericke University Magdeburg, Germany; 3 Institute of Diagnostic and Interventional Radiology, Hanover Medical School, Germany; 4 Faculty of Computer Science, University of Koblenz-Landau, Germany

**Keywords:** augmented reality, data visualisation, medical image processing, biomedical MRI, needles, augmented reality visualisation, needle guidance, MRI-guided interventions, operating field, displays, positions, patient, hand–eye coordination, needle navigation aids, needle puncture, projector-based augmented reality, visual feedback

## Abstract

During MRI-guided interventions, navigation support is often separated from the operating field on displays, which impedes the interpretation of positions and orientations of instruments inside the patient's body as well as hand–eye coordination. To overcome these issues projector-based augmented reality can be used to support needle guidance inside the MRI bore directly in the operating field. The authors present two visualisation concepts for needle navigation aids which were compared in an accuracy and usability study with eight participants, four of whom were experienced radiologists. The results show that both concepts are equally accurate ($2.0 \pm 0.6$ and $1.7 \pm 0.5\, {\rm mm}$), useful and easy to use, with clear visual feedback about the state and success of the needle puncture. For easier clinical applicability, a dynamic projection on moving surfaces and organ movement tracking are needed. For now, tests with patients with respiratory arrest are feasible.

## 1 Introduction

During minimally invasive interventions, target lesions are accessed through small entry points with instruments such as laparoscopes, catheters or needle applicators.

Because the human body is not opened for such treatments, radiological images are required to locate target lesions, surgical instruments and risk structures. Many needle-based interventions are thus carried out with the help of ultrasound (US) or computed tomography (CT) scanners [1, 2]. US is widely available, compact and cheap, but does not reach deeper structures, especially under bones [3]. In contrast, CT images represent the whole operating area, but with limited soft tissue contrast [4] and only by using harmful ionising radiation. As an alternative, MRI does not emit ionising radiation and provides excellent soft tissue contrast, so that target lesions invisible for US or CT can be identified. In addition, image planes in oblique orientations can be acquired and morphologic as well as functional information (temperature changes, blood flow, diffusion) can be monitored. When using tracked instruments that are calibrated with the MRI the live imaging planes can even be aligned along the needle to always get the view of the needle's surroundings [5].

**Fig. 1:**
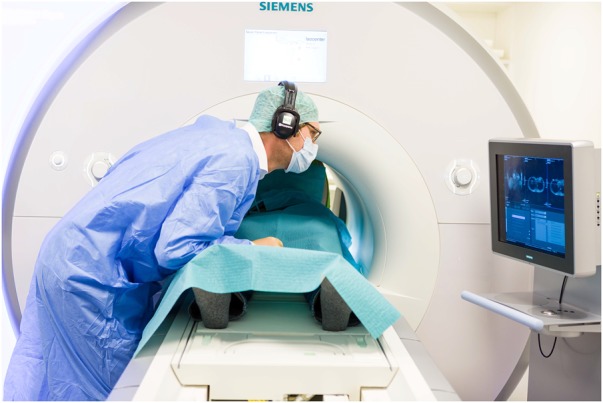
Current situation: A radiologist performs an MRI-guided needle puncture while using a separate display outside the MRI bore

To improve interventional MRI, appropriate instrument guidance is essential to simplify and shorten the intervention. Such assistance is often used on a display [6] (as shown in Fig. 1), which separates the useful information from the patient and increases mental load [7]. To overcome this issue, AR can be used to fuse the separate virtual data directly to the operating field.

Different Augmented Reality (AR)-based needle guidance systems have been introduced for the interventional MRI before. These include a head mounted display (HMD) to augment the radiologist's view of the patient with two-dimensional (2D) anatomical images and a virtual needle to navigate to the target [8], and a semi-transparent mirror placed in front of the MRI to visualise an overlay of 2D images and a needle navigation overlay [9]. Both techniques have the drawback in common that they require the patient to be translated out of the MRI because the HMD is not fully MR safe and the mirror occupies too much space to fit inside the bore. This interrupts the workflow and prevents the use of most of the MRI's advantages mentioned above, first and foremost live imaging, which is required to control the precise position of the needle during the puncture.

This work is based on a projector-based AR system for interventional MRI that is described in [10]. Based on this, we propose an AR visualisation approach that provides needle guidance information directly in the operating field inside the MRI bore. We present different concepts for AR needle navigation visualisation, their prototypical implementation and an assessment of their usability and accuracy.

## 2 State-of-the-art

Supporting needle guidance during image-guided interventions is a widely discussed topic. Separating the needle insertion task into three subtasks (tip positioning, needle alignment, needle insertion) and applying a cross-hair visualisation for needle positioning/alignment as well as a progress bar indicating the needle depth has been proposed [11, 12] and is nowadays implemented in many commercial navigation systems. Another approach is to overlay a video stream with the preview visualisation of the needle into the proper position that the user needs to target [6]. Because it is difficult to interpret navigation data relating to the patient on separate monitors, spatial AR approaches have emerged. One of them is a handheld projector augmenting arbitrary surfaces with explicit navigation aids and a needle path preview [13]. Alternatively, depth hints can also be given as concentric circles, where the inner expands to the outer circle with increasing depth, as described in [14]. Here, the needle orientation was led by an arrow. Besides projector-based AR, optical see-through AR using HMDs [8, 15] or head-up displays [16, 9, 17] are proposed. In both cases, the operating field was augmented with 2D images, needle positioning aids and basic distance cues. The latter plays a crucial role in AR applications. Especially in the medical context, it is important to correctly perceive the distance to risk structures to prevent injuries. Different visualisation approaches have been proposed to improve depth perception in AR, such as adaptive alpha blending on volumetric representations [18] or focus-and-context methods that consider the surrounding structures, i.e. bones and skin, for rendering and to create a window-like effect while angle and distance to the focused target objects are taken into account for transparency calculation [19].

Other approaches try to optimise the visualisation of 3D models themselves, in order to improve the perception of spatial relations and distances. Pseudo-chromadepth, a rendering technique where distance is represented by colours that follow the visible spectrum of light, was shown to improve relative depth perception [20]. Furthermore, illustrative visualisation encodes the shape and distance of vascular structures with different styles of hatching lines and can support depth perception in AR environments as well [21]. Similar approaches were presented by Wang *et al.* [22], who applied contour enhancing, occlusion and depth colour-coding to improve the perception of vascular structures.

In addition, augmented reality in the form of auditory feedback has been proposed to guide needles [23]. Bork *et al.* [24] combined both auditory and visual feedbacks to encode distance information between a surgical instrument and regions of interest. Their method includes a propagating shape around the instrument tip, which increases in size over time. Acoustic signals emphasise the growth process and speed of that shape. However, acoustic feedback may only be of limited use because of the noise from the MRI device.

## 3 Methods

To support needle guidance inside the interventional MRI, we developed two visualisation concepts. In order to accurately display navigation aids for needle insertion tasks directly on a patient, several steps were necessary. These steps are described in the following section. The navigation concepts are presented afterwards.

### 3.1 AR hardware setup

**Fig. 2:**
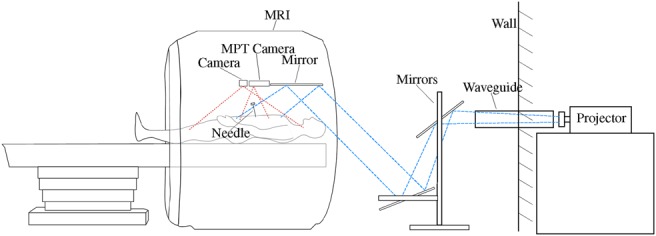
Schematic hardware setup of the augmented reality projector system

The basis of our navigation approach is a projector-camera system set up in the MRI scanning room. The long-throw projector with a resolution of 1024 × 768 px is located outside the room and its light is directed into the MRI bore via a waveguide through the wall and three mirrors (see Fig. 2). The detailed setup of the AR system, the extraction of the patient geometry, and the calibration process are described in [10].

For needle guidance applications, it is possible to track instruments with a commercial MPT system that is calibrated with the MRI. Therefore, three moiré phase markers are attached to a needle and calibrated with a tracked calibration body. Due to the fixed focus of the MPT camera, the tracking rate ranges from 115FPS, depending on the distance to the markers.

The projector is used to 3D scan the arbitrarily shaped projection surface via a structured-light approach. The result is a semi-dense point cloud of the projection surface which can then be further processed. The process of 3D scanning and generating the point cloud takes about 8 s in total. Then, a surface mesh is created from the point cloud that is used for later intersection calculations.

### 3.2 Needle interaction

In each frame, the needle's position and orientation are compared to the initially set needle path. The needle's intersection with the surface is determined by tracing the needle through the surface triangle mesh. The resulting intersection point is then compared with the planned needle insertion point. As a measure for the needle's orientation, the angle between the needle's direction vector and the vector between insertion and target point is calculated. This data will then be visualised depending on which visualisation concept is used.

### 3.3 Navigation by explicit aids (2D)

**Fig. 3:**
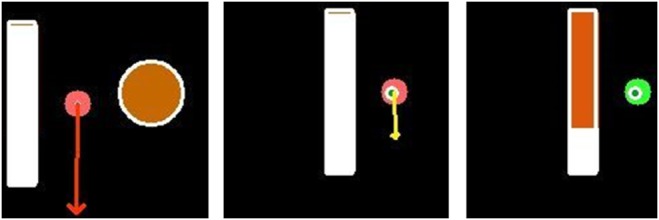
Visualisation of 2D explicit navigation aids. The visualisation contains the needle-surface intersection (red dot), the planned insertion point (orange/green circle with white borders), an arrow for needle alignment (red/yellow arrow) and a depth progress bar (red bar with white borders). After rendering, the visualisation is projected on the patient. Left: Needle is positioned next to the planned insertion point. Middle: Needle is positioned as planned (insertion point got smaller and green) and almost correctly aligned ($ \lt 1^\circ $; arrow turned yellow). Right: Needle is positioned and aligned as planned (arrow turned into a green sphere) and has already been inserted into the body

The first concept uses explicit aids to guide the user through the single insertion tasks positioning, alignment and insertion, as proposed in [11] and is illustrated in Fig. 3. The positioning of the needle at the planned insertion point is supported by a circle visualisation of adaptive size. The nearer the user positions the needle to the specified position, the smaller the radius of the circle. This is further clarified by a change of colour from red (high distance) to green (small distance).

The alignment process is supported by displaying an arrow originating at the insertion point and pointing towards the direction in which the needle shaft has to be tilted. The length of the arrow results from the angle between needle direction and designated needle path. The smaller the angle gets, the shorter the arrow is drawn. The length is logarithmically interpolated so that alignment changes at smaller differences to the planned orientation have less effect on the arrow length than changes at larger angle differences. That way, the arrow remains visible at small alignment deviations and thus allows fine adjustments. At an angular difference below 1°, the arrow's colour changes from red to yellow indicating an acceptable alignment. Further reducing the deviation below 0.5° causes the arrow to be replaced by a green dot, thus signalling a successful needle alignment.

Finally, the insertion step is supported by the visualisation of a progress bar. The filling of the bar is linearly dependent on the Euclidean distance between the needle tip and target structure. The bar begins filling up after inserting the needle. Reaching a distance below 0.4 mm, the bar's colour turns from red to yellow. After further reducing that distance to 0.2 mm, the progress bar switches its colour to green, thus implying a successful needle insertion. Over inserting will cause the colour to change back to red and continue downwards from the progress bar.

Due to the flat visualisation of explicit aids, this concept will be called concept 2D in the following.

### 3.4 Navigation by virtual vision through the skin (3D)

**Fig. 4:**
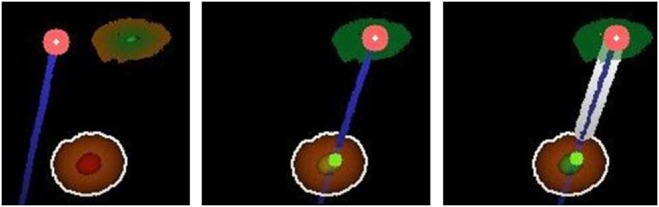
Navigation support by the display of virtual target and needle as is projected onto the operating field. The visualisation contains the needle-surface intersection (upper red dot), the virtual needle elongation (dark blue line), the planned insertion point (upper red/green circle) and the target (orange/red sphere). Left: Needle is positioned next to the planned insertion point. Middle: Needle is positioned as planned (insertion point is coloured green) and almost correctly aligned ($ \lt 1^\circ $; inner target zone turned yellow). Right: Needle is positioned and aligned as planned (inner target zone turned green) and was inserted into the body. The depth of the needle is indicated by a thick white border around the virtual blue needle

The second implemented approach enables the user to virtually see through the projection surface by visualising segmented structures, the aimed target and the needle inside the body. The concept is illustrated in Fig. 4. Those visualisations are rendered during a ray tracing process and show structures perspectively correct from the fixed viewing position. For each pixel, a ray is traced through the scene using the world coordinate correspondences. These rays are then checked for intersection with the surface triangle mesh and the cylinder representing the needle and its path. The resulting depth values at the intersection sites are then compared to initially calculated depth maps of the static objects. The depth values are then sorted and a pixel colour is computed with respect to the intersected objects’ colour and transparency values. The resultant colour of an object is the product of a set base colour, an applied Phong shading coefficient and a depth encoding coefficient. The latter is a linearly interpolated factor that depends on the distance to the viewing position. The higher the distance, the smaller the coefficient and the darker the final colour. Because the final image is displayed on the scene using a projector, dark colours cannot be visualised. Therefore, darkening colours results in applying transparency to them.

In order to support the user while using this concept, the needle is displayed virtually elongated as a cylinder. That way, the user can see the path the needle would follow if it was inserted at that moment. A small dot along the path line signifies where the needle would intersect an object. That dot's colour thereby depends on the kind of intersected object. Red illustrates the intersection of risk structures while green signalises the intersection of the target structure.

Furthermore, the target region is displayed as a two-zone sphere. That way, the target is visualised big enough to be quickly perceived while the inner zone emphasises the target's centre more precisely. The two zones are used for colour coding as well. The inner zone's colour represents the state of the needle's planned alignment and is equivalent to the previously described colours of the alignment arrow. The outer zone's colour represents the Euclidean distance from the needle tip to the target's centre and is coloured similarly to the previously described progress bar. A perfectly aligned needle at the target's centre thereby results in a target sphere with two green zones.

In addition, this concept uses a different approach to visualise the planned insertion point. The point is illustrated by a circle on the projection surface with a constant radius. The border of the circle is coloured red, while its centre is coloured green. The space in between is interpolated between these colours. The nearer the needle is positioned to the insertion point, the greater the green colour of the centre is weighted. A perfectly positioned needle results in a totally green circle. To further emphasise that, the colour lights up when the needle is positioned correctly.

**Fig. 5:**
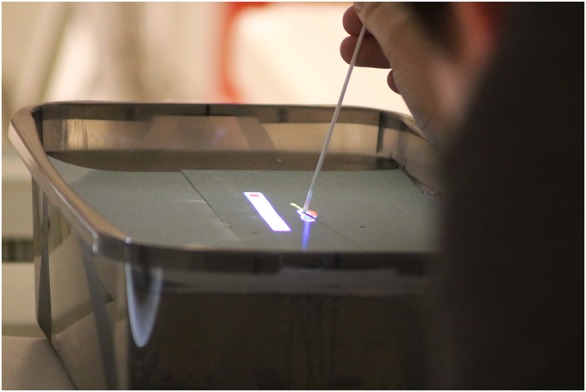
Exemplary view over the user's shoulder of the AR needle navigation inside the MRI bore. A combination of both 2D and 3D concepts is shown

This concept makes use of the visualisation of 3D structures inside the body. Therefore, this concept will be called concept 3D in this work. A real-world view of the needle navigation visualisation on a phantom is shown in Fig. 5. For demonstration purposes, the image shows both visualisation concepts.

## 4 Evaluation

In order to find out if the navigation concepts are useful and accurate, we conducted a user study with eight participants, four of whom were radiologists with 4–15 years of experience with minimally invasive needle-based image-guided interventions and four of whom were inexperienced users with a technical background in the medical domain. The users had the task to insert a tracked needle into a phantom body inside the MRI bore without image guidance, relying only on the AR interface described in this paper. The users were also told to keep their heads in the approximate predetermined viewing position because no eye tracking could be used to keep track of the correct viewpoint. However, the space inside the bore is very limited so that the users were not able to move the head around considerably. The needle needed to be positioned on the pre-planned entry point, aligned as desired and then inserted until the target was reached. Each user performed the task three times for each concept individually with different entry points and targets.

The phantom consisted of an opaque box filled with candle gel, which provided haptics similar to skin and other soft tissue and can be seen in a T1 MRI sequence dataset. In addition, rings were embedded in the candle gel as symbolic targets, which are visible in the MRI images. However, they were covered with a paper tissue so that the users were not influenced. The order of the used visualisation concepts was varied between the subjects via the biased coin method: The concepts were assigned to the two sides of the coin. It was flipped for the first user to determine the order of concepts, e.g. 3D first, then 2D. The second user then performed the tasks in the opposite order. The coin was flipped for every second user.

We measured the puncture duration as well as errors between the planned and reached entry and target positions in a post-operatively acquired dataset with the MRI's built-in distortion correction to best preserve the validity of the 3D positions. After each concept, each user answered the meCUE [25] and Nasa RawTLX [26] questionnaires so that insight into usefulness, usability, intention of use and subjective workload were given.

## 5 Results

The study did not reveal substantial differences between the concepts nor between experienced and inexperienced users (see Table 1) regarding positioning errors or puncture duration. Besides the duration of the insertion, there were no notable differences between user groups with regard to accuracy, subjective workload or user experience. Therefore, the results of the two concepts for all users are presented in the following. The subjective workload score from RawTLX for concept 2D (33.5) is slightly lower than for concept 3D (38.1), but still very similar. Frustration is higher with 3D (65.6) than with 2D (35), but mental demand was higher with 2D (37.5) than with 3D (25.6). The meCUE scores for both concepts are shown in Table 2.

**Table 1:** Results of the accuracy and duration measurements grouped by experience and concept

Experience	Concept	Puncture duration, s	Entry point error, mm	Target distance error, mm
med	2D	$127.8 \pm 45.4$	$2.1 \pm 0.9$	$1.7 \pm 0.5$
	3D	$96.60 \pm 41.2$	$1.9 \pm 1$	$1.5 \pm 0.4$
tech	2D	$85.25 \pm 30.9$	$1.9 \pm 0.4$	$2.3 \pm 0.5$
	3D	$106.5 \pm 33$	$1.4 \pm 0.3$	$1.8 \pm 0.6$
all	2D	$106.6 \pm 43.8$	$2.0 \pm 0.7$	$2.0 \pm 0.6$
	3D	$101.6 \pm 36.9$	$1.7 \pm 0.8$	$1.7 \pm 0.5$

**Table 2:** meCUE scores for both visualisation concepts. All scales are measured on a 7-point Likert scale, except the overall rating, which is from negative 5 to positive 5

scale	2D	3D
usefulness	$5.0 \pm 0.8$	$5.0 \pm 0.6$
usability	$5.6 \pm 1.0$	$5.5 \pm 1.0$
intention of use	$4.0 \pm 0.9$	$4.0 \pm 0.7$
overall	$1.9 \pm 0.6$	$2.8 \pm 0.9$

## 6 Discussion

We successfully demonstrated and tested two projector-based AR visualisation concepts for navigating a tracked needle from a planned entry point to a target position inside an MRI bore during minimally-invasive interventions. The accuracy measurements serve as an indicator of the quality of the registration as well as the operability. The values for the target point errors (2D: $2.0 \pm 0.6$, 3D: $1.7 \pm 0.5$) are comparable to those in the related literature ($1.8 \pm 1.5\, {\rm mm}$ [27], $4.0 \pm 12\, {\rm mm}$ [28], $2.2 \pm 06\, {\rm mm}$ [29], $2.2 \pm 07\, {\rm mm}$ [30], $1.1 \pm 05\, {\rm mm}$ [8]). The achieved accuracy can differ in a clinical setup due to needle deflection, which highly depends on the needle length and stiffness. During our experiments, the needle stiffness was very high and did not cause considerable deviations from the calibrated tip position with regard to the needle marker. Because of the virtual malformation of MRI scanned objects when processing the received signals, i.e. during the pre- and post-operative scans used for measurements, and the low resolution, these error values are inherently faulty and can only serve as an indication for accuracy.

The visualisation of the navigation aids and the virtual needle was perceived as perspective-correct even though it was fixed at an assumed viewing position. Nevertheless, the user's viewing position should be tracked in the future to guarantee a correct perspective and to make use of motion parallaxing and to thus create a more immersive visual representation of 3D objects.

The low MPT rate caused frustration with the users. Sometimes, when the MPT markers were too close to the MPT camera, the tracking stopped and the users did not have any feedback on the position or orientation of the needle. This led to more correcting movements, i.e. pulling the needle back and inserting it again, in both concepts. In addition, according to the RawTLX results, concept 3D shows a higher potential for frustration, because the users could not aim for the target as precisely as with concept 2D. Thus, many users had to correct more punctures as compared with concept 2D. However, the accuracy of both concepts and the usability and usefulness scores are similar, thus no superior concept can be determined.

Because some users commented they were more comfortable with one concept than with the other, it should remain the user's choice which one to use. Furthermore, no notable difference between the two user groups could be found in the study regarding the accuracy or subjective perception of workload or usability. This could be due to the easy-to-understand and unambiguous visualisations, which do not presume medical experience, as some users commented. The clear feedback during the whole process with colour and shape changes of the visual aids compensates for the professional differences.

By tracking the user's head position further development steps could enable kinetic depth cues by interactively adapting the visualisation to the viewing position. In addition, further illustrative visualisation approaches could be used to better encode depth information [21]. These additional depth cues could improve user performance and reduce frustration by better clarifying the 3D virtual scene. This navigation information should be visualised alongside explicit navigation aids and could thereby serve for visual inspection of the inserted needle position.

In total, the users confirmed with high meCUE usability and usefulness scores, as well as in comments, that the proposed needle guidance visualisation concepts serve as a suitable support for MRI-guided interventions. As suggested by the intention of use, the users would use this AR system beyond the study scope. To achieve clinical applicability the radiologists commented that organ movement tracking with ultrasound could be integrated and visualised and a dynamic surface registration to maintain position correctness of the projection is required. In the current state of the system, needle punctures could only be carried out during the artificially induced respiratory arrest of the intubated patient based on a planning dataset that is acquired breath triggered. The strength of the AR projection system shows during the first stage of a puncture when the needle can already be oriented accurately outside the body so that only small corrections are required when approaching the target. Thus, less healthy tissue will be damaged. This makes it also valuable for teaching radiologists.

## 7 Conclusion

In this work, we introduced two concepts for visualising needle guidance aids via a projector-based AR system inside the MRI bore directly in the operating field. In the 3D concept, a virtual depth-encoded needle, the planned entry point, and the target are projected. The virtual needle is elongated to facilitate the correct orientation in relation to the target. Changing colours reflect the current state as well as the correctness of the puncture. The 2D concept provides no 3D objects. Instead, the planned entry point, an orientation indicating arrow, and a progress bar showing the current distance to the target are visualised. A user study with four experienced radiologists and four participants with a technical background in the medical domain did not reveal a superior concept. Furthermore, no differences between the user groups could be found in terms of accuracy or usability assessment. This confirms that the visualisations are easy to use with clear feedback that can be understood without prior experience with the AR needle guidance system.

In summary, the proposed projector-based AR visualisation concepts represent a new approach to facilitate needle guidance for interventional MRI procedures, and the performed evaluation provides valuable insights. Further studies in experimental operating rooms and in the clinical environment are necessary to further improve the visualisation concepts and to evaluate their clinical applicability.

## 8 Funding and declaration of interests

This work is funded by the Federal Ministry of Education and Research (BMBF) within the STIMULATE research campus (grant no. 13GW0095A) and the German Research Foundation (grant nos. HA 7819/1-1 and LA 3855/1-1). Frank Wacker declares grants from Siemens Healthineers outside of this work.

## 9 Conflict of interest

None declared.
